# Efficacy and Safety of PARP Inhibitors in Advanced or Metastatic Triple-Negative Breast Cancer: A Systematic Review and Meta-Analysis

**DOI:** 10.3389/fonc.2021.742139

**Published:** 2021-10-28

**Authors:** Xu Liu, Kan Wu, Dan Zheng, Chuanxu Luo, Yu Fan, Xiaorong Zhong, Hong Zheng

**Affiliations:** ^1^ Laboratory of Molecular Diagnosis of Cancer, Clinical Research Center for Breast, West China Hospital, Sichuan University, Chengdu, China; ^2^ Department of Urology, Institute of Urology, West China Hospital, Sichuan University, Sichuan, China; ^3^ Department of Head, Neck and Mammary Gland Oncology, Cancer Center, West China Hospital, Sichuan University, Chengdu, China

**Keywords:** metastatic triple-negative breast cancer, PARP inhibitor, *BRCA* mutation, homologous recombination deficiency, efficacy, safety

## Abstract

**Purpose:**

Poly (ADP-ribose) polymerase (PARP) inhibitors have shown promising results in metastatic triple-negative breast cancers (TNBCs). We therefore performed a systematic review and meta-analysis to evaluate the efficacy and safety of this drug in patients with advanced or metastatic TNBC.

**Methods:**

A systematic literature search of PubMed, Embase, Scopus, Web of Science, and Cochrane Central Register of Controlled Trials for synonyms of “PARP inhibitors” and “breast cancer” was carried out. All published phase II/III clinical studies of PARP inhibitors in patients with advanced/metastatic TNBC were screened. Data were extracted independently by two authors and analyzed using Review Manager software version 5.3. End points include overall response rate (ORR), progression-free survival (PFS), and adverse events.

**Results:**

Ten clinical trials were identified, with a total of 1,495 patients included. Pooled analyses showed that PARP inhibitors could provide a significant improvement of ORR [risk ratio (RR) = 2.00; 95% confidence interval (CI), 1.14–3.50; p = 0.02) and PFS [hazard ratio (HR) = 0.68; 95%Cl, 0.59–0.77; p < 0.0001) compared to chemotherapy in the whole population. In subgroup analysis, patients with *BRCA* mutation had a higher objective response to PARP inhibitor, with an RR of 2.85 (95%CI, 1.34–6.06; p = 0.007) compared to *BRCA* wild-type patients. However, no significant difference in ORR was observed between the homologous recombination deficiency (HRD) positive and non-HRD subgroups (RR = 1.82; 95%CI, 0.81–4.08; p = 0.14). Hematological toxicity is a common adverse event of PARP inhibitors.

**Conclusions:**

PARP inhibitors are effective options for the treatment of patients with advanced or metastatic TNBC. Compared with patients without germline *BRCA* mutation, patients with germline *BRCA* mutation could benefit more from PARP inhibitors. In clinical setting, hematological toxicity associated with PARP inhibitors should be monitored regularly.

## Introduction

Triple-negative breast cancer (TNBC) is an aggressive subtype of breast cancer, accounting for 15%–20% of all cases of breast cancer (1, 2). Cytotoxic chemotherapy is currently the main treatment of patients with TNBC due to the lack of expression of estrogen receptor, progesterone receptor, and human epidermal growth factor receptor 2 (3). Unfortunately, TNBC often develops resistance to chemotherapy and eventually progresses to lethal metastatic disease, with a median survival of approximately 1 year (4, 5). Notably, more than one-third of TNBC patients will develop distant metastases (6), and there is no standard of care therapy for patients with metastatic TNBC (mTNBC) (7). Therefore, there is an urgent need to develop new therapeutic approach for patients with mTNBC.

With the advancement of genomics analysis, new druggable targets are being identified, which may contribute to broadening novel therapeutic scenario for mTNBC. Among patients with TNBC, about 10%–30% cases present with germline *BRCA*1 or *BRCA*2 gene mutation (8, 9). On the other hand, approximately 70% of *BRCA*1-mutated breast cancers have a triple-negative phenotype (10). *BRCA*1 and *BRCA*2 genes are involved in the homologous recombination repair (HRR) pathway and are responsible for repairing DNA double-strand breaks. The HRR pathway also contains many other genes, such as *ATM*, *PALB2* , *RAD51*, *CDK12*, and *CHK1/2*. Alterations on these genes can lead to homologous recombination deficiency (HRD). Poly (ADP-ribose) polymerase (PARP) is an important enzyme for repairing DNA single-strand break (11, 12). Thus, the inactivation of PARP in tumors with HRD will increase genomic instability and eventually result in cell death. Preclinical studies have shown that cancer cells with functional *BRCA*1 or *BRCA*2 mutations are sensitive to PARP inhibitors (13), providing a strong rationale for using PARP inhibitors to treat mTNBC.

There are currently several PARP inhibitors being tested in clinical trials in patients with mTNBC. Based on the promising results observed in clinical trials, olaparib has been approved by the Food and Drug Administration (FDA) for the treatment of patients with germline *BRCA* mutation (gm*BRCA*) and HER2-negative metastatic breast cancer (14, 15). Several clinical trials have shown that PARP inhibitors could also confer a survival benefit in patients with metastatic TNBC irrespective of *BRCA* status (16, 17). Therefore, in this systematic review and meta-analysis, we aimed to comprehensively evaluate the efficacy and safety of PARP inhibitors in patients with advanced or metastatic TNBC based on available clinical trial results. We also explored biomarkers to determine the subgroup of patients who could benefit most from PARP inhibitors.

## Methods and Materials

### Search Strategy

On August 2020, a systematic literature search was performed by two independent reviewers through PubMed, Embase, Scopus, Web of Science, and Cochrane Central Register of Controlled Trials according to the Preferred Reporting Items for Systematic Review and Meta-analysis (PRISMA) guidelines (18). The search terms are as follows: [(“Poly(ADP-ribose) Polymerase inhibitors” OR “PARP inhibitors”] OR “Olaparib” OR “rucaparib” OR “talazoparib” OR “veliparib” OR “niraparib” OR “iniparib”) AND (“breast”) AND (“randomized controlled trial” OR “clinical trial”).

### Inclusion and Exclusion Criteria

The following were the inclusion criteria: (1) phase II and III clinical trials evaluating the efficacy of PARP inhibitor as single agent or in combination with other anticancer drugs in the treatment of patients with advanced or metastatic TNBC were considered for inclusion; (2) the eligible studies mentioned objective response rate (ORR), progression-free survival (PFS), or safety outcomes; and (3) only English-language articles were included.

Exclusion criteria were as follows: (1) phase I clinical trial, case reports, editorials, review articles, and retrospective studies were excluded; (2) single-arm studies that did not report *BRCA* or HRD status were not included; (3) clinical trials focusing on neoadjuvant therapy; (4) finally, for the continuously updated and published follow-up data, the latest results were considered for analysis. The selected studies were identified based on inclusion and exclusion criteria by two independent reviewers.

### Data Extraction

Two authors independently extracted data from eligible studies included in the meta-analysis. The following data were included: first author’s information, year of publication, study design, trial phase, ClinicalTrial.gov Number, sample size, *BRCA* or HRD status, type of intervention/control, efficacy results (ORR and PFS), and numbers of adverse events (AEs) in each arm. If the PFS was only represented by the Kaplan–Meier curve, the Engauge digitizer 4.1 software was used to digitize and extract the data [only in one study (19)].

### Risk of Bias Assessment

The potential risk of bias in the selected studies was independently assessed by two reviewers, using Cochrane Risk of bias tool, which included selection bias, performance bias, detection bias, attrition bias, reporting bias, and other possible sources of bias. The risk of bias was graded as high, low, or unclear risk. Disagreements were resolved through consensus or a third reviewer.

### Study Objectives

The objective of this study was to compare the antitumor efficacy and safety between the PARP inhibitor group and the chemotherapy group. The primary outcomes of this meta-analysis were ORR and PFS; AEs were the secondary outcomes. We also performed exploratory subgroup analyses to investigate the therapeutic activity of PARP inhibitors in the *BRCA*-mutated group *vs. BRCA* wild-type group, and HRD group *vs.* non-HRD group.

### Statistical Analysis

The hazard ratio (HR) with its 95% confidence interval (CI) was calculated to compare the PFS. The risk ratios (RR) and 95% CI were calculated to measure the ORR and AEs. A two-sided p-value <0.05 was considered statistically significant. The statistical heterogeneity was assessed using the I^2^ statistic and chi-squared test. When heterogeneity was observed (I^2^ > 50% and p < 0.05), the random effects model was applied; otherwise, the fixed effects model was used. Funnel plot was used to detect potential publication bias. All statistical analyses were performed using Review Manager software version 5.3.

## Results

### Study Selection and Characteristics

**Figure 1:**
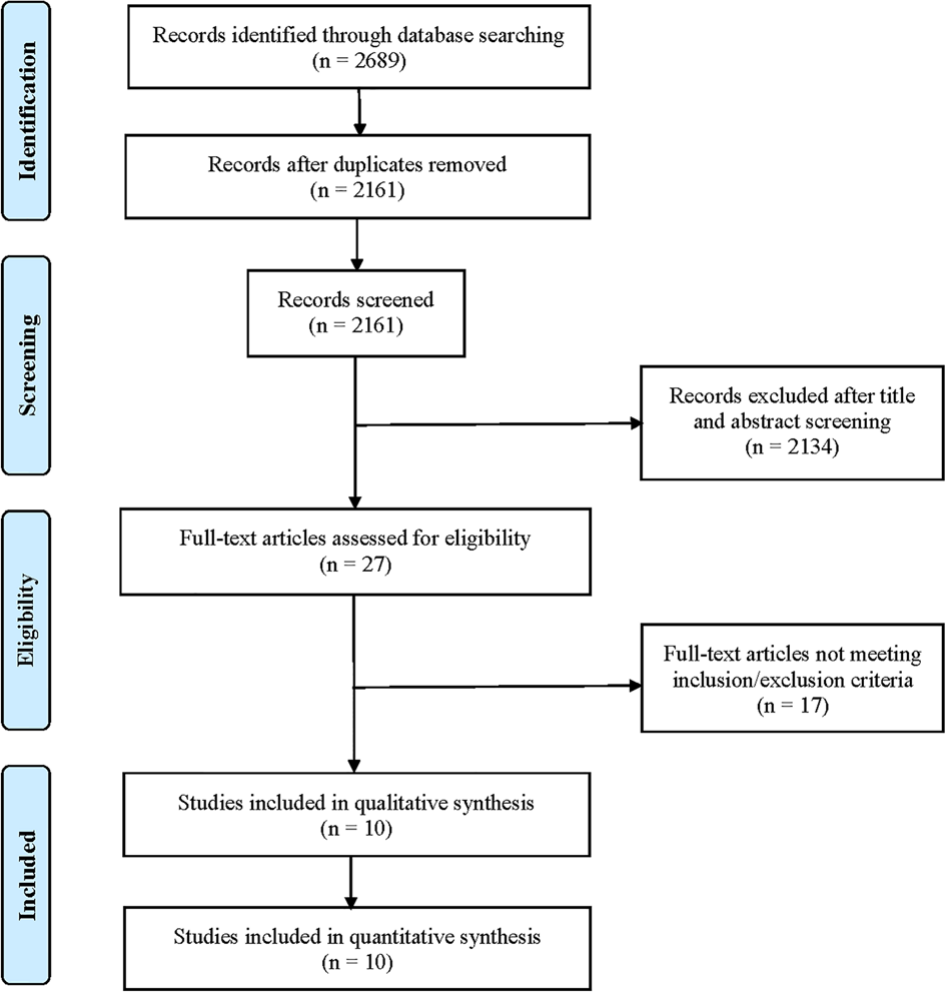
Flow diagram of study inclusion and exclusion.

After searching the electronic databases, a total of 2,689 records were initially retrieved (**Figure 1**). After removing duplicates and screening titles and abstracts, only 27 full-text articles were further assessed for their eligibility based on inclusion/exclusion criteria. After full-text review, 17 published articles were excluded for the following reasons: 5 articles did not report relevant outcomes of this study population; 5 studies reported results from the same population; 4 were clinical trials of neoadjuvant therapy for TNBC; 2 single-arm studies did not report HRD or *BRCA* mutation status; and 1 single-arm study only reported *BRCA* mutations. Finally, 10 clinical trials were included for final pooled analysis, including 7 randomized controlled trials (14, 16, 17, 19–22) and 3 single-arm studies (23–25). Of the 10 studies, 8 studies reported *BRCA* mutation status. Among them, the type of *BRCA* mutation reported in five studies was germline mutation, two studies were germline or somatic *BRCA* mutations, and the type of *BRCA* mutation reported in another study was unclear. For the BROCADE study (21), they randomly set up two comparison groups: veliparib with carboplatin/paclitaxel *versus* placebo plus carboplatin/paclitaxel, and veliparib plus temozolomide *versus* placebo plus carboplatin/paclitaxel; we only evaluated veliparib plus carboplatin/paclitaxel *versus* placebo plus carboplatin/paclitaxel to avoid statistical influences on research weight.

The main features of selected studies and enrolled patients are summarized in **Table 1**. All clinical trials reported the antitumor efficacy of PARP inhibitors in patients with advanced or metastatic TNBC, ranging from 21 to 519 patients per study. Globally, a total of 1,495 patients were included in the meta-analysis, of whom 735 patients harbored somatic or germline *BRCA1/2* mutations.

**Table 1:** Characteristics of the selected studies.

Study (Year)	Study Name (NCT number)	Phase	Study design	Treatment	Total no. of TNBC patients	No. of *BRCA*mut patients	No. of *BRCA*wt patients	No. of HRD patients
Gelmon et al. (23)	NCT00679783	II	Single-arm	Olaparib	21	5	16	NA
O’Shaughnessy et al. (16)	NCT00540358	II	RCT	Iniparib + gemcitabine and carboplatin *vs* gemcitabine and carboplatin	123	NA	NA	NA
O’Shaughnessy et al. (17)	NCT00938652	III	RCT	Iniparib + gemcitabine and carboplatin *vs* gemcitabine and carboplatin	519	NA	NA	NA
Kummar et al. (19)	NCT01306032	II	RCT	Veliparib + cyclophosphamide *vs* cyclophosphamide	45	7	4	NA
Robson et al. (14)	OlympiAD NCT02000622	III	RCT	Olaparib *vs* standard therapy	150	150	0	NA
Litton et al. (20)	EMBRACA NCT01945775	III	RCT	Talazoparib *vs* standard single-agent therapy	190	190	0	NA
Han et al. (21)	BROCADE NCT01506609	II	RCT	Veliparib + carboplatin/paclitaxel *vs* placebo + carboplatin/paclitaxel	120	120	0	NA
Vinayak et al. (24)	TOPACIO NCT02657889	II	Single-arm	Niraparib + pembrolizumab	55	15	27	20
Shimomura et al. (25)	EO UMIN000018721	II	Single-arm	Olaparib + Eribulin	29	5	24	9
Diéras et al. (22)	BROCADE3 NCT02163694	III	RCT	Veliparib + carboplatin-paclitaxel *vs* placebo + carboplatin/paclitaxel	243	243	0	NA

NCT, ClinicalTrials.gov identifier; TNBC, triple-negative breast cancer; BRCAmut, BRCA mutation; BRCAwt, BRCA wild type; HRD, homologous recombination deficiency; NA, not applicable.

### Efficacy of PARP Inhibitors in Advanced/Metastatic TNBC

#### PARP Inhibitors *vs.* Control

**Figure 2:**
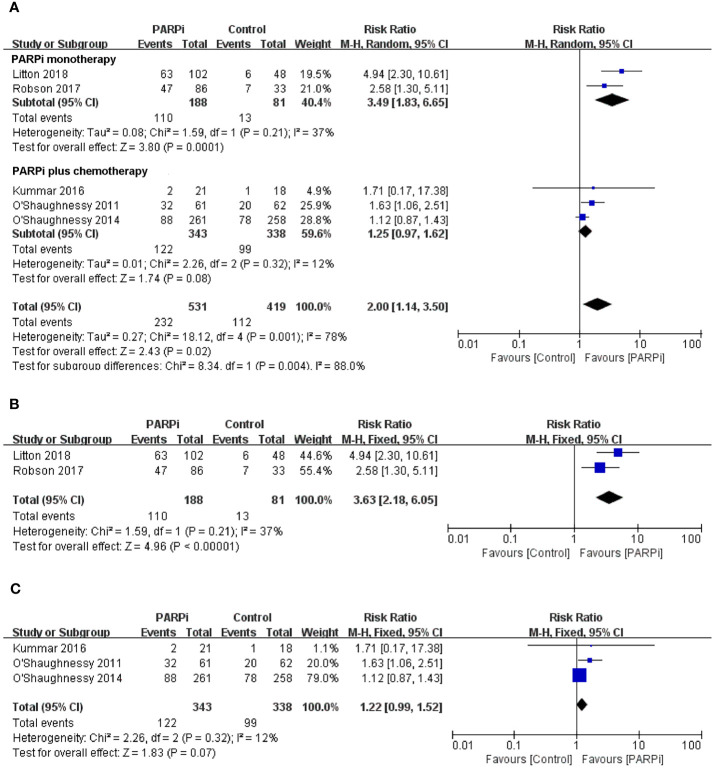
Forest plots of pooled analyses for PARP inhibitors *vs* control treatment on objective response rate in **(A)** total patients, **(B)** *BRCA*-mutated patients, and **(C)** unselected patients.

**Figure 3:**
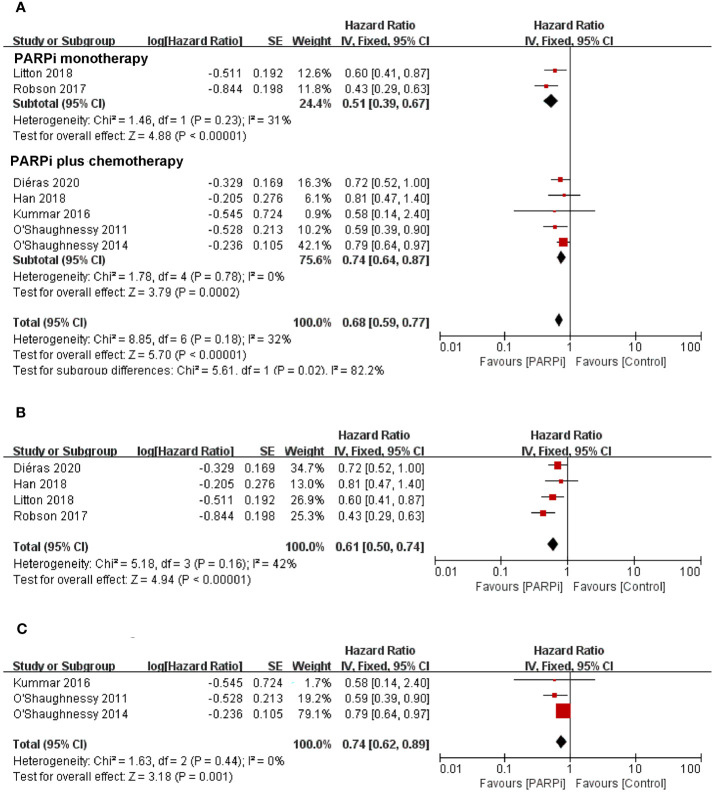
Forest plots of pooled analyses for PARP inhibitors *vs*. control treatment on progression-free survival in **(A)** total patients, **(B)** *BRCA*-mutated patients, and **(C)** unselected patients.

To evaluate the effect of PARP inhibitors on patients with advanced/metastatic TNBC, we first conducted a pooled analysis to compare the antitumor efficacy between the PARP inhibitor group and the chemotherapy group. Seven randomized controlled trials were pooled into the analysis of ORR or PFS (14, 16, 17, 19–22), including 778 patients with advanced/metastatic TNBC who received PARP inhibitors (olaparib, iniparib, veliparib, or talazoparib) and 568 participants who were administered with chemotherapy. In two studies, PARP inhibitors were investigated as monotherapy against standard chemotherapy. In five studies, PARP inhibitors were investigated in combination with cyclophosphamide, gemcitabine-carboplatin, and carboplatin/paclitaxel and compared with placebo. In the whole population, the pooled RR showed that PARP inhibitor treatment significantly improved the incidence of achieving ORR compared to chemotherapy (RR = 2.00; 95% CI, 1.14–3.50; p = 0.02) (**Figure 2A**). The pooled analysis for PFS indicated that the PARP inhibitor group had a better PFS (HR = 0.68; 95% CI, 0.59–0.77, p < 0.0001) when compared with the control group whether in combined therapy or monotherapy [HR = 0.74, 95% CI (0.64–0.87); HR = 0.51, 95% CI (0.39–0.67), respectively] (**Figure 3A**).

With regard to the clinical benefit of PARP inhibitors in patients with *BRCA* mutations, four randomized controlled trials were eligible for the analysis of ORR or PFS (14, 20–22). The above studies showed that compared with chemotherapy, PARP inhibitor treatment significantly improved ORR (RR = 3.63; 95% CI, 2.18–6.05; p < 0.0001) (**Figure 2B**) and PFS (HR = 0.61; 95% CI, 0.50–0.74; p < 0.0001) in the patients with *BRCA* mutation (**Figure 3B**).

Three clinical trials focused on the efficacy of PARP inhibitors in advanced or metastatic TNBC irrespective of *BRCA* or HRD status (16, 17, 19); we performed a pooled analysis in this unselected population. The results showed that no significant difference in ORR was observed between the PARP inhibitor group and the chemotherapy group (RR = 1.22; 95% CI, 0.99–1.52; p = 0.07) (**Figure 2C**). However, PARP inhibitors showed a significant improvement in PFS (HR = 0.74; 95% CI, 0.62–0.89; p = 0.001) (**Figure 3C**).

### Subgroup Analysis of the Efficacy of PARP Inhibitors

#### *BRCA* Mutated *vs*. *BRCA* Wild Type

**Figure 4:**
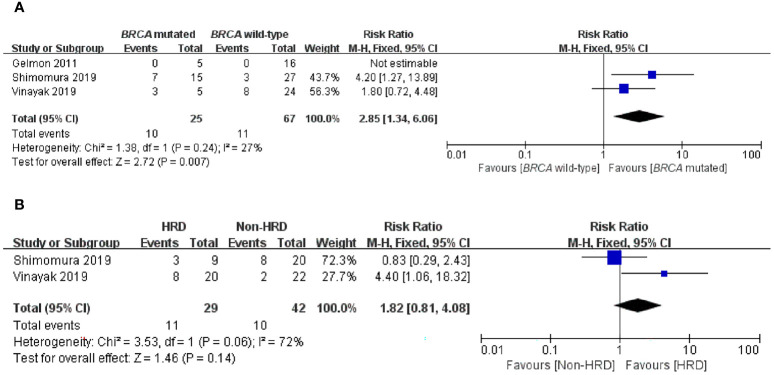
Forest plots of pooled analyses for the effect of PARP inhibitors on ORR in **(A)** *BRCA*-mutated *vs*. *BRCA* wild-type patients and **(B)** HRD *vs*. non-HRD patients.

To further compare the efficacy of PARP inhibitors in the *BRCA*-mutated and *BRCA* wild-type populations, we subsequently conducted an exploratory analysis that directly compared these two groups. Three studies mentioned ORR in two subgroups and were incorporated into this analysis (23–25). Subgroup analysis demonstrated that PARP inhibitors could provide a significant improvement in ORR to patients with *BRCA* mutation in comparison to patients without *BRCA* mutation, with an RR of 2.85 (95% CI, 1.34–6.06; p = 0.007) (**Figure 4A**).

#### HRD *vs*. Non-HRD

It is still unclear whether, in addition to *BRCA* mutations, HRD status can be used as a biomarker for predicting PARP inhibitors sensitivity in an advanced/metastatic TNBC setting. Therefore, we performed a subgroup analysis of the HRD-positive group *vs*. the non-HRD group to address this question. Two articles were eligible for this analysis, and only ORR data were available in the two studies with low statistical power (24, 25). Interestingly, when comparing the HRD-positive subgroup with the non-HRD subgroup, there was no significant difference in ORR (RR = 1.82; 95% CI, 0.81–4.08; p = 0.14) (**Figure 4B**).

### Adverse Events of PARP Inhibitor

In this study, seven randomized controlled trials reporting AEs were used for risk analysis. The comparative safety profile in terms of the AEs of interest is shown in **Table 2**. On the whole, the results showed that the incidence of AEs in the PARP inhibitor group was similar to that in the chemotherapy group, regardless of any grade AEs (98.94% *vs*. 98.98%; RR = 1.00; 95% CI, 0.99–1.01; p = 0.66) and grade ≥3 AEs (76.32% *vs*. 79.68%; RR = 0.97; 95% CI, 0.88–1.07; p = 0.54). Notably, PARP inhibitors slightly increased the overall risk of serious AEs compared with chemotherapy (26.88% *vs*. 24.57%; RR = 1.18; 95% CI, 1.00–1.38; p = 0.05). In the PARP inhibitor group, for any grade of events, the five most common AEs were nausea (64.00%), neutropenia (60.30%), thrombocytopenia (59.20%), anemia (59.17%), and fatigue (51.70%). For grade ≥3 AEs, they were neutropenia (47.03%), thrombocytopenia (30.32%), anemia (27.56%), leukopenia (14.78%), and fatigue (5.13%). The pooled data showed that compared with the chemotherapy group, the PARP inhibitor group had an increased incidence of AEs in terms of grade ≥3 thrombocytopenia (p < 0.001), any grade nausea (p < 0.001), and any grade vomiting (p = 0.04).

**Table 2:** Summary of the adverse events (AEs).

Adverse events	No. of studies	Adverse events/total patients (%)		RR	95% CI	p-value
		PARP inhibitors	Control treatment			
Any grade adverse events	6	1,311/1,325 (98.94)	779/787(98.98)	1.00	0.99–1.01	0.66
Grade ≥3 adverse events	6	809/1,060 (76.32)	541/679 (79.68)	0.97	0.88–1.07	0.54
Any grade neutropenia	6	799/1,325 (60.30)	533/787 (67.73)	0.90	0.78–1.03	0.12
Grade ≥3 neutropenia	7	633/1,346 (47.03)	430/805 (53.42)	0.85	0.69–1.05	0.13
Any grade anemia	6	784/1,325 (59.17)	407/787 (51.72)	1.22	0.97–1.54	0.09
Grade ≥3 anemia	7	371/1,346 (27.56)	158/805 (19.63)	1.52	0.86–2.67	0.15
Any grade thrombocytopenia	5	663/1,120 (59.20)	359/696 (51.58)	1.19	0.99–1.43	0.06
Grade ≥3 thrombocytopenia	6	346/1,141 (30.32)	149/714 (20.87)	1.50	1.26–1.77	<0.001
Any grade leukopenia	6	346/1,325 (26.11)	209/787 (26.56)	0.95	0.82–1.10	0.51
Grade ≥3 leukopenia	7	199/1,346 (14.78)	121/805 (15.03)	1.00	0.81–1.22	0.98
Any grade fatigue	6	685/1,325 (51.70)	415/787 (52.73)	1.05	0.97–1.14	0.22
Grade ≥3 fatigue	6	68/1,325 (5.13)	44/787 (5.59)	1.04	0.72–1.51	0.83
Any grade nausea	6	848/1,325 (64.00)	439/787 (55.78)	1.17	1.09–1.26	<0.001
Grade ≥3 nausea	6	30/1,325 (2.26)	21/787 (2.67)	0.87	0.49–1.55	0.64
Any grade constipation	5	406/1,120 (36.25)	245/696 (35.20)	1.12	0.99–1.27	0.08
Grade ≥3 constipation	5	6/1,120 (0.54)	3/696 (0.43)	1.19	0.35–4.06	0.78
Any grade vomiting	6	424/1,325 (32.00)	224/787 (28.46)	1.16	1.01–1.33	0.04
Grade ≥3 vomiting	6	32/1,325 (2.42)	11/787 (1.40)	1.69	0.87–3.29	0.12
Any grade diarrhea	6	394/1,325 (29.74)	214/787 (27.19)	1.08	0.94–1.24	0.29
Grade ≥3 diarrhea	6	32/1,325 (2.42)	22/787 (2.80)	0.82	0.49–1.37	0.44
Any grade decreased appetite	4	215/1,013 (21.22)	105/484 (21.69)	0.99	0.80–1.21	0.89
Grade ≥3 decreased appetite	4	7/1,013 (0.69)	2/484 (0.41)	1.49	0.36–6.13	0.58

### Quality of Included Studies

The “risk of bias graph” revealed that this meta-analysis had a moderate risk of selection bias because 3 out of 10 clinical trials were single-arm studies (**Supplementary Figure S1**). We used the funnel plots to detect publication bias, and the results suggested that there was a relatively low risk of publication bias (**Supplementary Figure S2**).

## Discussion

The results of this study highlight that compared with chemotherapy, PARP inhibitors can safely and significantly improve ORR and PFS in patients with advanced/metastatic TNBC. Furthermore, exploratory analysis showed that the patients with mutation in *BRCA* could derive more benefits from PARP inhibitors than *BRCA* wild-type patients. However, we did not observe any difference in tumor control between HRD-positive patients and non-HRD patients. Based on these recent clinical evidence, *BRCA* mutations, rather than HRD status, can be used as a predictive biomarker of response to PARP inhibitors in the advanced/metastatic TNBC setting.

Both *BRCA*1 and *BRCA*2 genes are key components of the HRR pathway and are responsible for repairing DNA double-strand breaks. Alterations on *BRCA*1/2 or other components may lead to HRD. PARP is an important enzyme in response to DNA single-strand breaks. Inhibition of PARP can cause DNA single-strand breaks, which subsequently results in DNA double-strand breaks. In normal cells, these breaks can be repaired through the HRR pathway. However, in HR-deficient tumors, the breaks caused by PARP inhibition would not be repaired, eventually leading to tumor cell death (26). Preclinical studies have shown that PARP inhibitors have greater efficacy in *BRCA*-deficient cells when compared with wild-type cells (27, 28). In a clinical setting, a proof-of-concept trial by Tutt showed that PARP inhibitor treatment has a favorable therapeutic index for patients with germline *BRCA* mutation and advanced breast cancer (29). Based on these results, over the past years, several clinical trials have been conducted and are currently evaluating the effects of different PARP inhibitors in this population. Specifically, olaparib has been approved for patients with a germline *BRCA* mutation and HER2-negative metastatic breast cancer based on the results of the OlympiAD study (14). In view of this meta-analysis, our conclusion also confirmed that patients with germline *BRCA* mutation might be prime candidates for PARP inhibition treatment. However, our analysis also found that PARP inhibitors could provide significant improvement in PFS for unselected patients, regardless of *BRCA* mutational status. Similarly, in phase II and III clinical trials of patients with metastatic TNBC, irrespective of *BRCA* status (16, 17), the gain in PFS was obtained in the PARP inhibition group compared with the chemotherapy group. In the setting of neoadjuvant treatment, PARP inhibitors could also increase the pathological complete response rate of TNBC population, regardless of *BRCA* status (30). Moreover, those with *BRCA* mutation account for only a small proportion of breast cancer patients (31). Hence, using only *BRCA* status as a predictive biomarker of PARP inhibitors sensitivity is insufficient, and many potential responders may be missed.

The main question facing oncologists is how to go about practically selecting patients with advanced/metastatic TNBC who will benefit from PARP inhibitor therapy. With large-scale sequencing analysis, in addition to *BRCA1/2* gene, many other HRD genes (*ATM*, *CHK1/2*, and *PTEN*) were found to be correlated with PARP inhibition sensitivity, which could be utilized as alternative biomarkers for identifying susceptible population (32–34). In clinical situations, HRD status has good predictive power for the benefits of PARP inhibitors in several cancer types, such as ovarian, prostate, and gastric cancer (35). However, in this study, we did not observe any improvement in ORR of HRD-positive patients compared with HRD-negative patients. It should be noted that there were only two relevant studies in this subgroup analysis, with small sample size and low statistical power, making it difficult to draw strong conclusions. In addition, recent studies have shown that low *RAD51* score, high TIL, or high PDL1 expression are all associated with the response to PARP inhibitors in TNBC, which indicates that in addition to *BRCA*ness signature, immunological features could be regarded as potential predictive biomarkers of PARP inhibitors (36–38). In the future, more clinical trials are needed to evaluate the predictive value of HRD in the TNBC setting and to explore new biomarkers for determining optimal patients who are more likely to benefit from PARP inhibitors.

As PARP inhibitors have gradually been approved for clinical application worldwide, it is of great value and indispensable significance to evaluate the safety and tolerance of PARP inhibitors in patients. Our pooled analysis of 1,346 patients with advanced/metastatic breast cancer treated with PARP inhibitors from seven randomized controlled trials showed that the three most common AEs of grade 3 and above were neutropenia (47.03%), thrombocytopenia (30.32%), and anemia (27.56%), indicating that hematological toxicity events caused by PARP inhibition treatment are more common and serious. Similarly, a meta-analysis of eight clinical trials also found that olaparib could significantly increase the risk of severe neutropenia in cancer patients (39). This may be because PARP inhibitors not only interfere with the DNA repair of cancer cells but may also interfere with rapidly dividing blood cells, thus leading to myelosuppression. Notably, our meta-analysis showed that PARP inhibitors did not increase the incidence of grade ≥3 AEs except for the risk of thrombocytopenia when compared with chemotherapy. Overall, PARP inhibitors seem to be safe and tolerable for patients with advanced/metastatic breast cancer, but the high risk of PARP inhibitor-related hematological toxicity is an issue that cannot be ignored and should be considered in clinical applications.

There are several limitations in the present meta-analysis. First, the potential biases of this study included the heterogeneity of inclusion criteria, patients and treatment schedule in the included trials, for example, age, race, disease status, type of PARP inhibitors, and interventional arm. These confounding variables were not stratified properly and incorporated into the meta-analysis. In addition, there are few comparative studies on the efficacy of PARP inhibitors between HRD-positive patients and HRD-negative patients, which makes it difficult to fully assess the benefit of PARP inhibitors based on the HRD status of patients. Therefore, it is warranted to carry out randomized controlled trials with longer clinical follow-up time in the future.

## Conclusions

Our findings confirm that PARP inhibitor is an effective, well-tolerated treatment in patients with advanced/metastatic TNBC. We also support the view that *BRCA* status can be used as a predictive biomarker of PARP inhibitor sensitivity to guide clinical decision-making. However, the predictive value of HRD status still needs to be further evaluated in future studies. Hematological toxicity is a common adverse event; thus, regular hematological monitoring is warranted for patients receiving PARP inhibitors.

## Data Availability Statement

The original contributions presented in the study are included in the article/**Supplementary Material**. Further inquiries can be directed to the corresponding authors.

## Author Contributions

XL, KW, and XZ conceived and designed the analysis. XL and KW collected the data. XL, KW, and DZ analyzed the data. CL, YF, XL, and HZ interpreted the data. All authors were involved in the drafting, critical review, and approval of the final manuscript and the decision to submit for publication.

## Funding

This work was supported by the key program of the Science and Technology Department of Sichuan Province (grant number 2017SZ0005 to HZ); the 135 Project for Disciplines of Excellence, West China Hospital, Sichuan University (grant number ZYGD18012 to HB), and the 135 Project for Disciplines of Excellence, West China Hospital, Sichuan University (grant number ZYJC21035).

## Conflict of Interest

The authors declare that the research was conducted in the absence of any commercial or financial relationships that could be construed as a potential conflict of interest.

## Publisher’s Note

All claims expressed in this article are solely those of the authors and do not necessarily represent those of their affiliated organizations, or those of the publisher, the editors and the reviewers. Any product that may be evaluated in this article, or claim that may be made by its manufacturer, is not guaranteed or endorsed by the publisher.
