# Supplementary material for: Efficacy and Safety of PARP Inhibitors in Advanced or Metastatic Triple-Negative Breast Cancer: A Systematic Review and Meta-Analysis
Source: Front Oncol. 2021 Oct 28;11:742139. doi: 10.3389/fonc.2021.742139 (PMC8581463; doi:10.3389/fonc.2021.742139)
Supplement: Supplementary file 1 [file DataSheet_1.docx]

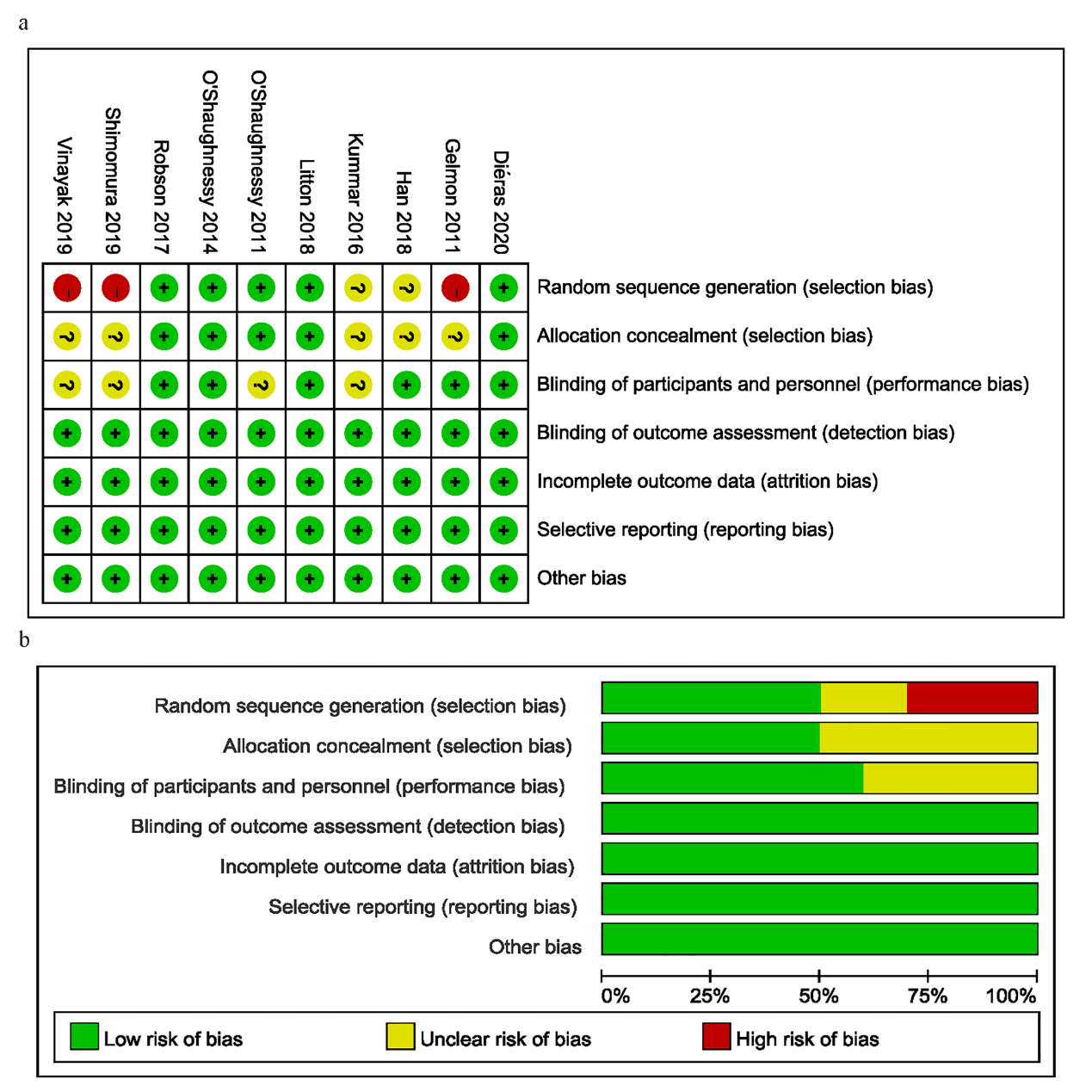


**Supplementary Figure. 1.** Risk of bias graph.

**
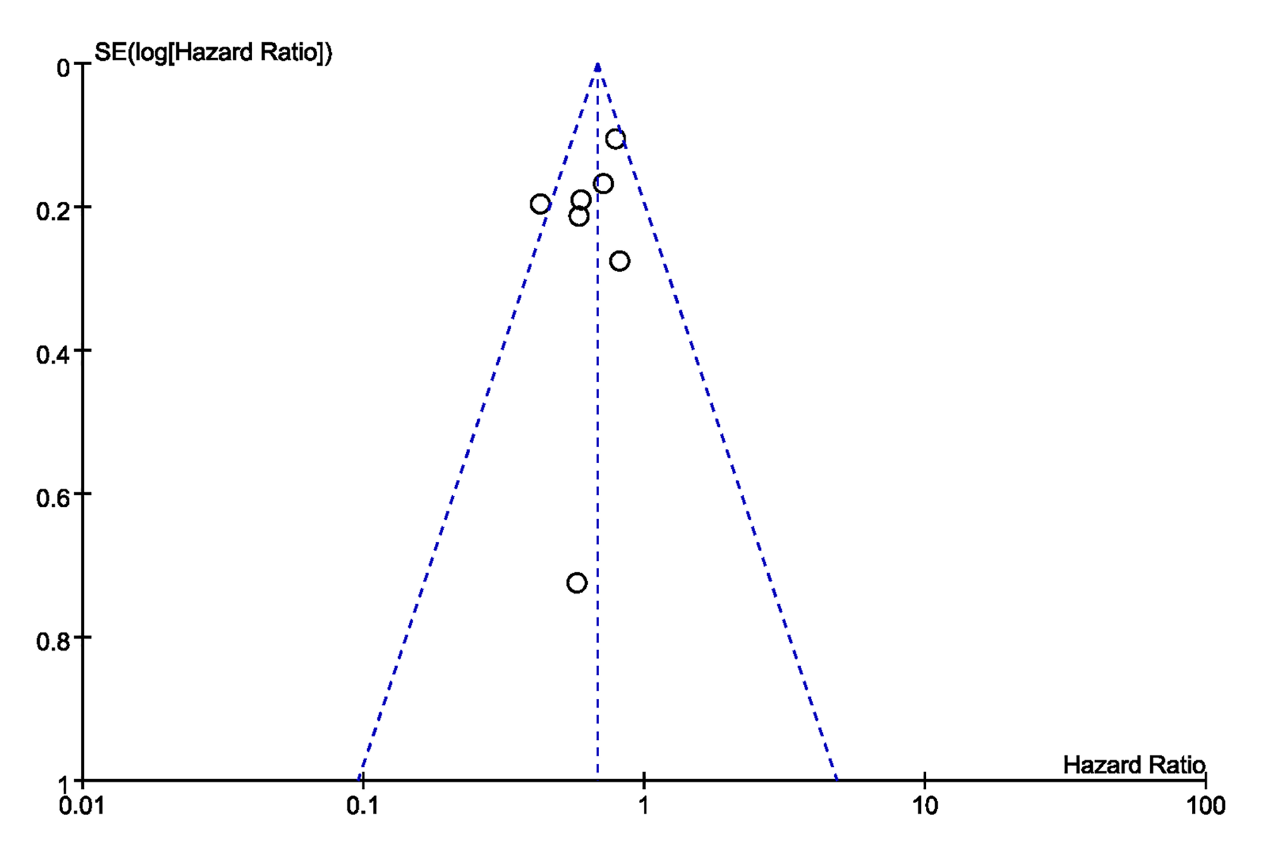
**

**Supplementary Figure. 2.** Funnel plot for all randomized controlled trials.
